# Continuous noninvasive hemoglobin monitoring estimates timing for detecting anemia better than clinicians: a randomized controlled trial

**DOI:** 10.1186/s12871-019-0755-1

**Published:** 2019-05-17

**Authors:** Bo Tang, Xuerong Yu, Li Xu, Afang Zhu, Yuelun Zhang, Yuguang Huang

**Affiliations:** Department of Anesthesiology, Peking Union Medical College Hospital, Chinese Academy of Medical Sciences, Peking Union Medical College, Beijing, China; No.1 Shuaifuyuan, Wangfujing Street, Beijing, 100730 China

**Keywords:** Pulse CO-oximetry, Hemoglobin, Monitoring, Anemia, Surgery, Trends

## Abstract

**Background:**

Hemoglobin measurement is important for transfusion decision-making. Pulse CO-Oximetry provides real-time continuous hemoglobin (SpHb) monitoring. The triage role of SpHb trends based on hemoglobin measurements was investigated.

**Methods:**

In this diagnostic randomized controlled trial, 69 patients undergoing spine or cytoreductive surgery were randomly enrolled into SpHb-monitoring and standard-care groups. Diagnostic blood samples were drawn for CO-oximetry Hb (CoOxHb) when the SpHb decreased by 1 g/dl or at the clinician’s discretion in the standard-care group. The positive predictive value (PPV) was defined as the ability to detect a decrease in CoOxHb > 1 g/dl or a CoOxHb < 10 g/dl; the PPVs were compared using Fisher’s exact test. The SpHb and trend accuracies were calculated. The transfusion units and postoperative hemoglobin levels were compared.

**Results:**

The PPV of a decrease in CoOxHb > 1 g/dl was 93.3% in the SpHb group vs 54.5% without SpHb monitoring (*p* = 0.002). The PPV of CoOxHb < 10 g/dl was 86.7% vs. 50.0% for these groups (*p* = 0.015). The CoOxHb was never < 7 g/dl with SpHb monitoring. Sixty SpHb–CoOxHb data pairs and 28 delta pairs (ΔSpHb-ΔCoOxHb) were collected. The bias, precision and limits of agreement were − 0.29, 1.03 and − 2.30 to 1.72 g/dl, respectively. When ΔSpHb and ΔCoOxHb were > 1 g/dl, the concordance rate for changes in hemoglobin reached 100%. The delta pairs revealed a positive correlation [ΔSpHb = 0.49 * ΔCoOxHb - 0.13; *r* = 0.69, 95% confidence interval (0.53, 0.82)]. No significant differences were found in the transfusion volume or postoperative anemia state.

**Conclusions:**

The SpHb trend tracked changes in hemoglobin satisfactorily during surgery and more accurately estimated the appropriate timing for invasive hemoglobin measurements than the clinicians.

**Trial registration:**

ChiCTR1800016290 (Prospective registered). Initial registration date was 24/05/2018.

## Background

A red blood cell transfusion decision should be made based on the patient’s hemoglobin concentration (Hb) to balance sufficient oxygen supply and transfusion-related reactions. In China, the rate of unnecessary erythrocyte transfusion reached 30.9, 95% CI [confidence interval] = 27.1–35.0% [1] and was 28.8% in our institution; this rate was higher in surgical departments than in other departments [2]. A high number of transfusions are performed without objective indications.

In the current clinical pathway for anemia detection during intraoperative blood loss, an invasive Hb measurement is performed at the clinicians’ discretion. Requiring clinicians to determine anemia is energy consuming and often inaccurate. Attention must be paid not only to the patient’s vital signs, blood volume in the surgical field, cotton pads, and cell savers but also ongoing hemostasis procedures. The discretion of the clinician is totally subjective and is largely dependent on their clinical experience

.Moreover, Hb measurements are often omitted during intraoperative blood loss. Traditional Hb measurements such as the auto analysis of blood cells and CO-oximetry analysis require blood samples, the collection of which is invasive, time-consuming and intermittent. Considering the absence of anesthesia nurses in most hospitals in China, anesthesiologists have to send the blood sample in person, which often results in delays or is omitted in favor of completing more important work. Consequently, transfusion is often performed without any objective indications, which may result in unnecessary blood transfusions in patients lacking the necessary indications or delayed blood transfusions in bleeding patients.

Pulse CO-Oximetry (Radical 7, Masimo, Irvine, CA, USA) is a multiwavelength spectrophotometric technique providing continuous, noninvasive monitoring of total Hb (SpHb). The method is based on measurement of the differential optical density of seven different wavelengths of light passing through the finger and has received Food and Drug Administration 510(k) clearance. Although SpHb monitoring is not sufficiently accurate to replace invasive measurements [3, 4], studies have demonstrated acceptable correlations between the trends in SpHb and Hb [5–9]. A study reported a strong ability of SpHb to reliably detect changes in CoOxHb > 1 g/dl [10]. SpHb may be able to inform physicians of decreases in hemoglobin concentrations in a timely and accurate manner, preventing unnecessary diagnostic blood draws and offering detailed clinical evidence for transfusion decisions during surgery.

The potential triage role of continuous, real-time monitoring of Hb stability should not be underestimated. The aim of this study was to investigate whether noninvasive, continuous and real-time monitoring of Hb could estimate the timing for further Hb measurements more accurately than clinicians’ discretion during surgery.

## Methods

### Participants

Through a departmental quality review of transfusion practices, patients undergoing spine surgery on multiple spinal segments and cytoreductive surgery were identified; these surgeries were more likely to be associated with a sufficiently large blood loss volume to trigger anemia. The inclusion criteria were patients aged from 18 to 80 years who were scheduled for spine surgery or cytoreductive surgery, for whom the estimated blood loss was more than 15% of their total blood volume and who required an arterial catheter as a part of the standard care procedures for continuous arterial pressure monitoring and intermittent blood analysis. The exclusion criteria were an American Society of Anesthesiologists physical status score > 3, an inability to use their upper extremities for SpHb monitoring, preoperative anemia (male laboratory vein Hb < 12 g/dl and female < 11 g/dl), hepatic insufficiency (phosphatase alkaline, aspartate aminotransferase, alanine aminotransferase > 2 times the normal values), impaired renal function (serum creatinine > 1.5 mg/dl), coagulation disorders (activated partial thromboplastin time > 1.5 times the normal value or taking antiplatelet/anticoagulation drug) and pregnancy.

### Interventions

#### SpHb monitoring group

In the SpHb monitoring group, an adhesive sensor (R2-25a), connected to the Radical-7® Pulse CO-Oximeter (software version V7740, Masimo Corp., Irvine, CA), was placed on the proximal third of the nail bed of the second, third, or fourth finger of the hand on the side opposite the arterial catheter before the induction of general anesthesia. The adhesive portion of the sensor was applied according to the directions for use and was covered with opaque shields to prevent optical interference. If the perfusion index, which is an indicator of localized perfusion, was < 1%, then the sensor position was recalibrated by switching the monitor off and on.

The baseline SpHb was recorded after the SpHb was stable for at least 15 min following induction of anesthesia. To obtain a time-matched invasive Hb concentration, a blood sample was drawn through the radial arterial catheter placed in the wrist contralateral to the SpHb sensor. Then, the CoOxHb was obtained using a CO-Oximeter (Radiometer ABL800; Radiometer, Copenhagen, Denmark), which is the routine method for intraoperative Hb measurement in our hospital. An SpHb level 1 g/dl lower than the baseline was set as the threshold for the alarm. When the SpHb monitor sounded the alarm or the surgery ended, both the SpHb and CoOxHb were recorded simultaneously. When clinicians deemed it necessary to take a Hb measurement but the pulse CO-Oximetry did not generate an alert, blood was drawn and tested to ensure the patient’s safety.

#### Standard care group

In the standard care group, the first blood sample was drawn from the radial arterial catheter and tested for CoOxHb at 20 min following the induction of anesthesia as the baseline. Other Hb measurements during surgery were ordered at the discretion of the clinicians.

Intraoperative and postoperative transfusion decisions in both groups were made by the clinicians considering both the CoOxHb value and the clinical situation.

### Primary and secondary outcomes

**Fig. 1:**
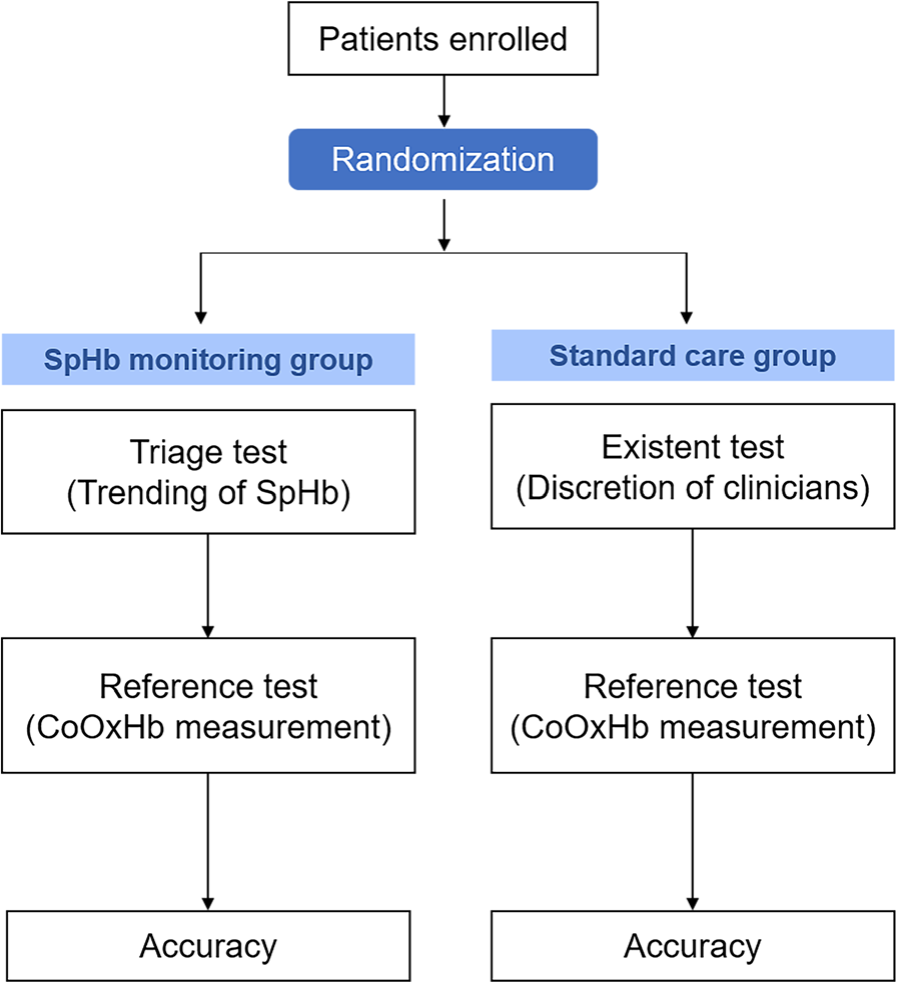
Flow chart of the diagnostic randomized controlled trial

The hypothesis of the study was that SpHb monitoring could more appropriately estimate the timing for further Hb measurement than clinicians during surgery without creating a delay in treatment. The primary outcomes were the positive predictive values (PPVs) of SpHb monitoring and the clinicians’ perception of the decrease in CoOxHb. A true-positive finding was defined as the ability to detect a decrease in CoOxHb of 1 g/dl or CoOxHb < 10 g/dl, whereas a false-positive finding was defined as an inability to detect stable CoOxHb (Fig. 1).

The secondary outcomes included a delay in treatment, the absolute accuracy and precision of SpHb monitoring compared with the reference method (CO-oximeter), and the accuracy of the trend in SpHb compared with changes in CoOxHb. A delay in treatment was defined as a decrease in CoOxHb to 7 g/dl, which was the intraoperative transfusion threshold in China.

### Sample size

The sample size calculation was performed using internet-based software (http://www.sample-size.net/) based on the comparison of two independent proportions using exact test. In our hospital, the PPV of the clinicians’ perception of a substantial decline (> 1 g/dl) in CoOxHb was estimated to be less than 50%. Based on the results of the pilot trial, the study was designed to raise the PPV from 50 to 90% with SpHb monitoring, assuming a power of 0.8 and a two-sided significance level (α) of 0.05. We calculated a required sample size of 20 per group. Considering that the incidence of the loss of a sufficient quantity of blood to lead to anemia during cytoreductive or spine surgeries was approximately 25%, we planned to enroll 160 patients in total (80 per group).

### Randomization, allocation and blinding

The trial was randomized (1:1) created using a website-generated (https://www.random.org/coins/) allocation list. Group allocations were concealed in sequentially numbered opaque envelopes, which were opened after the patients entered the operating rooms. After identifying the random number, the research nurse provided SpHb monitoring for the patient or offered standard care following the protocol.

The investigator who performed the statistical analysis was blinded to the group allocation, whereas the research nurse and anesthesiologists were not blinded.

### Statistical methods

Continuous variables are expressed as the means ± standard deviation (SD) when normally distributed and as interquartile ranges (25th to 75th percentile) when not normally distributed. The distribution of variables was checked using visual inspection of histogram. Variables were compared using the t test or Mann-Whitney U test when appropriate. Categorical variables are presented as numbers and percentages (%) and were compared using Fisher’s exact test.

In line with the primary objective, PPVs were presented as percentages and compared by Fisher’s exact test.

For the SpHb accuracy analysis, the Bland-Altman test for data pairs of SpHb and CoOxHb was used to compare bias (mean error), precision (standard deviation, SD) and limits of agreement (LOA) using MedCalc (version 18.11, MedCalc Software, Acacialaan, Ostend, Belgium). The agreement between the two assays was presented by data plotting.

For the SpHb trend accuracy analysis, a four-quadrant plot was generated to evaluate clinically significant directional changes [delta (Δ) Hb > 1 g/dl] and a regression analysis was performed to calculate correlation coefficients (r) and 95% CIs were reported for all data using R (version 3.5.1, The R Foundation, Welthandelsplatz, Vienna, Austria). The coefficient from the model could be interpreted as each unit increase of SpHb was associated with CoOxHb. A two-sided *p*-value less than 0.05 was considered statistically significant. Statistical analysis was carried out using IBM SPSS for Windows (version 22.0, IBM Corporation, Armonk, New York, USA).

## Results

**Fig. 2:**
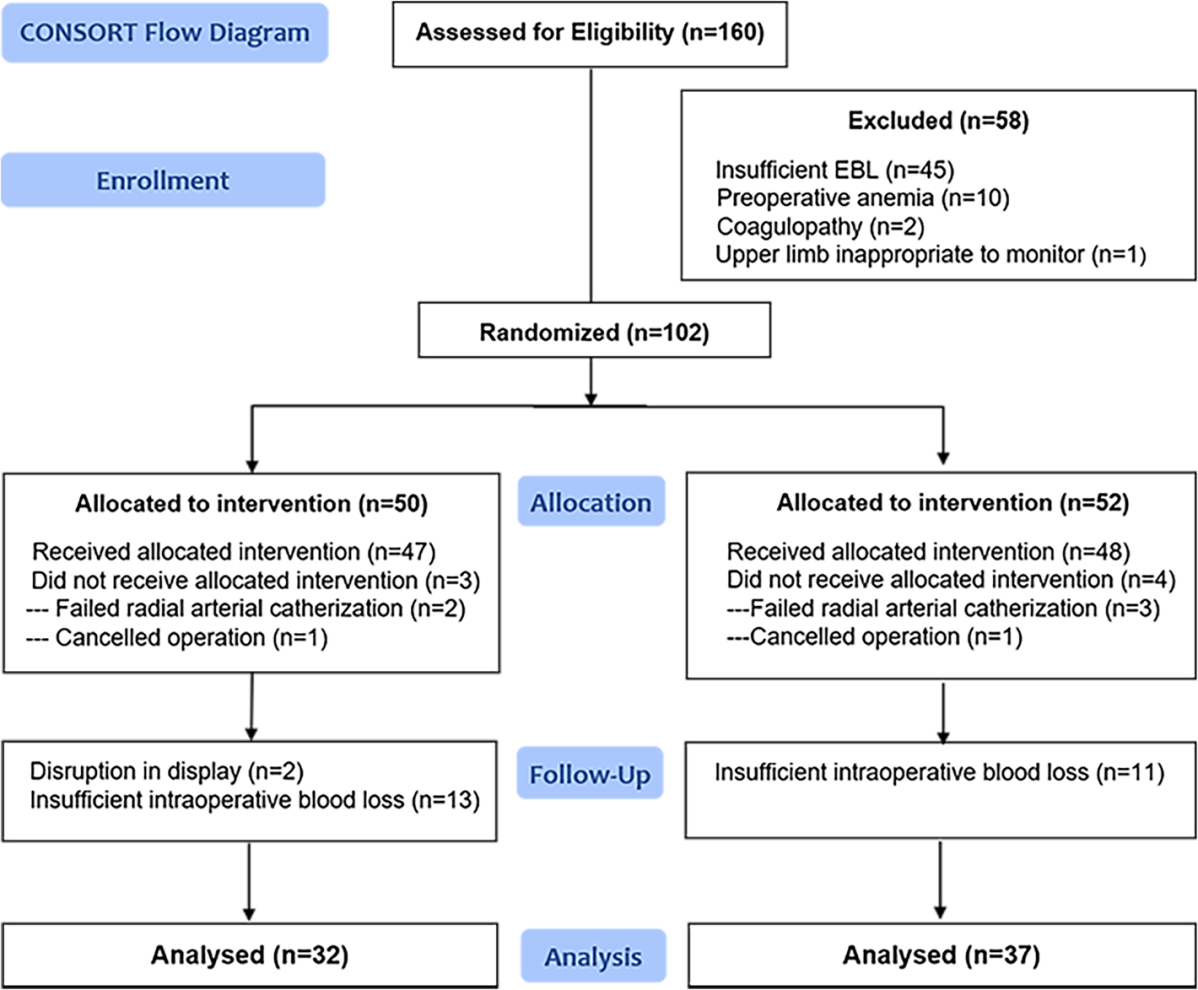
Consolidated Standards of Reporting Trials flow diagram

One hundred sixty patients were assessed for eligibility from May 2018 to September 2018, of whom fifty-eight patients were excluded before allocation for various reasons (Fig. 2). In total, 69 patients completed the protocol and were included in the analysis. The participant flow diagram is shown below. No case of injury was found during the surgical procedures. The participant characteristics, operation types and intraoperative blood loss were similar between the groups (Table 1).

**Table 1:** Demographic and surgery related characteristics in SpHb monitoring group and Standard care group

	SpHb monitoring group (*n* = 32)	Standard care group (*n* = 37)
Sex, n		
Women	26 (81.25%)	30 (81.08%)
Men	6 (18.75%)	7 (18.92%)
Age, y	51.5 ± 13.8	50.4 ± 14.4
Body weight, kg	61.8 ± 15.0	58.9 ± 10.4
Type of surgery, n		
Cytoreductive surgery	21 (65.63%)	26 (70.27%)
Spinal surgery	11 (34.37%)	11 (29.73%)
Preoperative hemoglobin, g/dl	11.4 ± 1.0	11.7 ± 1.2
Intraoperative blood loss, ml	600 (400 to 1000)	600 (400 to 1000)
Intraoperative blood loss over 15% of total blood volume, n	19 (59.38%)	22 (59.50%)
End of surgery, n		
Transferred to ICU	10 (31.25%)	13 (35.14%)
Back to ward	22 (68.75%)	24 (64.86%)

Data are expressed as the number of cases (%), the mean ± SD or the median (interquartile range). Abbreviations: *ICU* intensive care units

The incidence of unnecessary Hb measurement was lower in the SpHb monitoring group than in the standard care group. The PPV of a decrease in CoOxHb > 1 g/dl was 93.3% based on SpHb monitoring and 54.5% based on the clinicians’ perception (*p* = 0.002). The PPV of CoOxHb falling below 10 g/dl was 86.7% vs. 50.0% for these groups (*p* = 0.015). No case had a CoOxHb < 7 g/dl with SpHb monitoring (Table 2).

**Table 2:** Intraoperative invasive Hb results

	SpHb monitoring group	Standard care group	*p*
PPV of a decrease in CoOxHb > 1 g/dl	93.3%	54.5%	0.002
PPV of CoOxHb falling to 10 g/dl	86.7%	50.0%	0.015
CoOxHb < 7 g/dl, n	0	1	

Data are expressed as percentages or numbers of patients. PPVs were compared using Fisher’s exact test. *PPV* positive predictive value

**Fig. 3:**
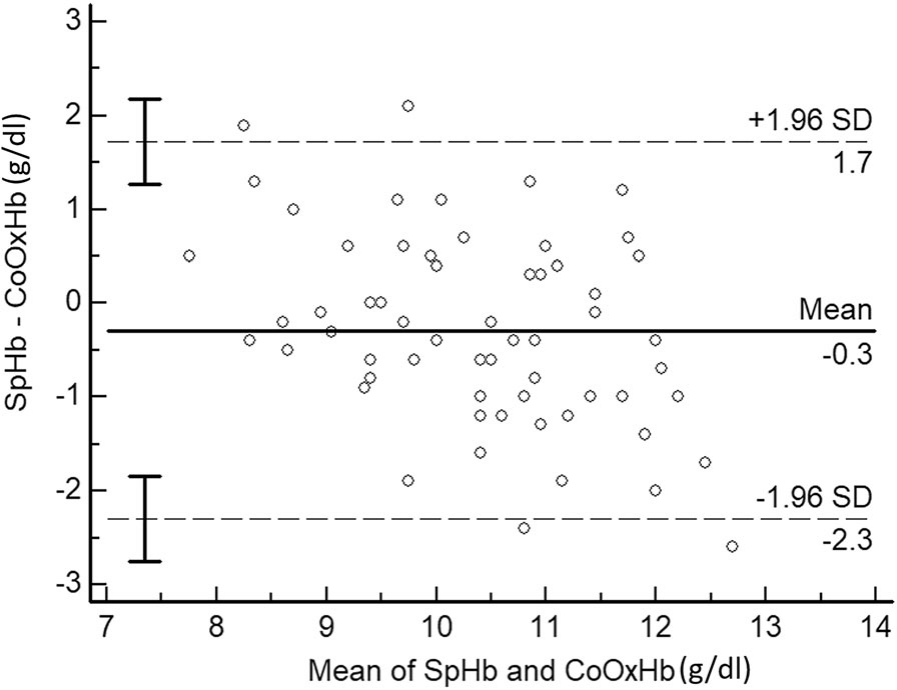
Bland-Altman analysis of SpHb and CoOxHb. Bland-Altman plot for comparison of differences in Hb values measured by pulse CO-Oximetry (SpHb) and CO-oximetry (CoOxHb) to the average Hb measurements from both methods (SpHb and CoOxHb). The dotted lines correspond to the 95% LOA corresponding to 1.96 SD

The absolute bias ± precision of SpHb monitoring compared with that of CoOxHb measurement was − 0.29 ± 1.03 g/dl, with LOAs = − 2.30, 1.72 g/dl according to the Bland-Altman analysis (Fig. 3).

**Fig. 4:**
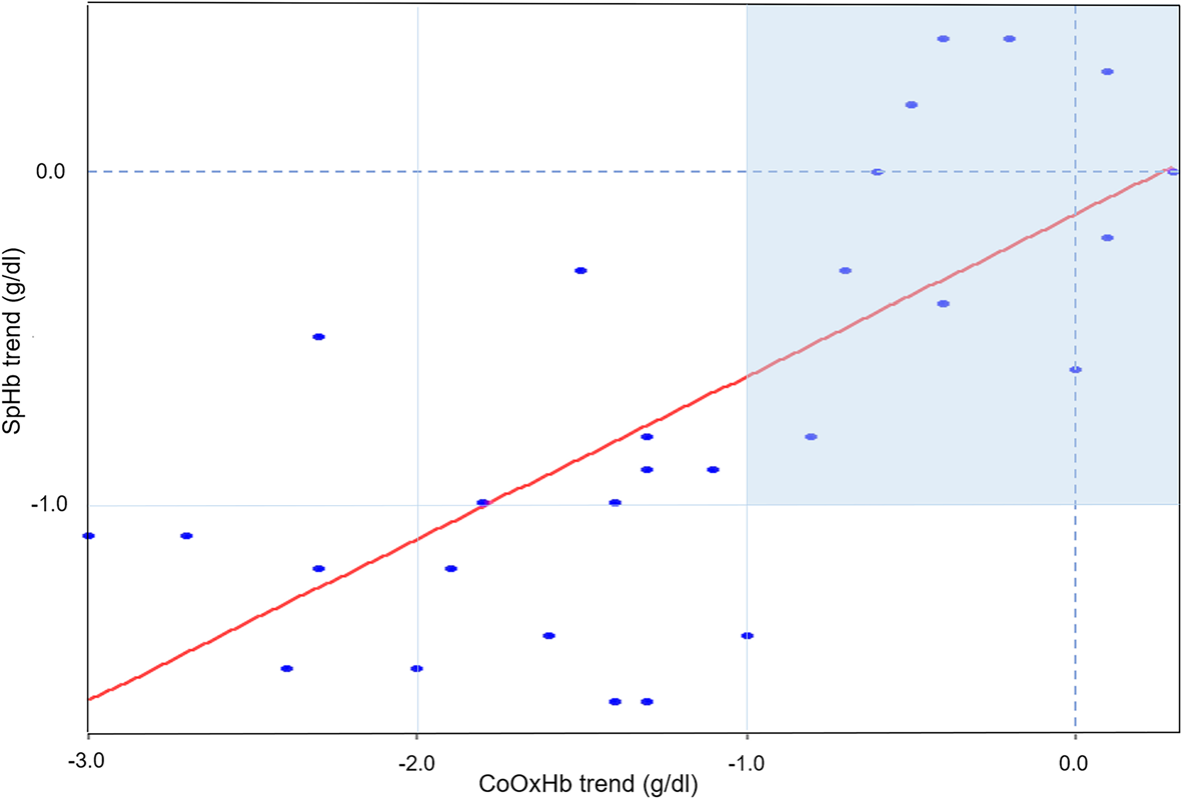
Four-quadrant plot for ΔSpHb and ΔCoOxHb. Four-quadrant graphical representation of changes in the absolute values of SpHb and CoOxHb (28 paired delta data points). The solid line indicates the regression line. Data pairs outside the dark area (exclusion zone) are over the clinically significant threshold of 1 g/dl

To evaluate the trends in ΔSpHb and ΔCoOxHb, 28 data pairs were collected and displayed a strong positive correlation, with *r* = 0.69, 95% CI = 0.53, 0.82 (*p* < 0.001). Linear regression analysis revealed ΔSpHb = 0.49*ΔCoOxHb - 0.13 (Fig. 4). In the four-quadrant plot, 17 data points were outside the exclusion zone of 1 g/dl and were located in the lower left quadrant, indicating appropriate changes in SpHb and CoOxHb in the same direction.

No difference was observed in the intraoperative transfusion units or postoperative Hb concentrations between the two groups (Table 3).

**Table 3:** Intraoperative transfusion units and postoperative Hb concentration

	SpHb monitoring group	Standard care group	*p*
Intraoperative transfusion units, n	0 (0 to 1)	0 (0 to 1)	0.938
CoOxHb on POD1, g/dl	10.8 (9.6 to 12.2)	11.0 (9.9 to 11.9)	0.705
CoOxHb < 10 g/dl on POD1, %	31.3%	24.3%	0.520
CoOxHb when discharged, g/dl	10.6 (9.8 to 11.1)	10.5 (9.9 to 11.3)	0.921

Data are expressed as the median (interquartile range) or proportion of cases. The median was compared using the Mann-Whitney U test and Fisher’s exact test for categorical variables. POD 1, the first postoperative day

## Discussion

In our study, a diagnostic randomized trial was designed to investigate the triage role of SpHb trends in the intraoperative detection of anemia. A decrease of 1 g/dl in SpHb was set as the indication for the further invasive hemoglobin test. Using an absolute change in CoOxHb of > 1 g/dl, a concurrent absolute change in SpHb of > 1 g/dl provided a positive a predictive value of 93.3%, which was much higher than the PPV estimated by clinicians (50%).

Pulse CO-Oximetry is a promising technique that provides noninvasive, continuous detection of anemia but cannot be used as a substitute for invasive Hb measurements. Multiple factors, such as serious anemia and large amounts of vascular drugs leading to poor peripheral perfusion [11, 12], can reduce the accuracy of SpHb monitoring. We also found the decreased accuracy during blood loss. Several studies have aimed to determine whether continuous monitoring of SpHb can warn clinicians of anemia [13]. John Hopkins Hospital demonstrated that the SpHb reached a threshold of 8 g/dl before the laboratory results in 44% of cases and was unable to identify the threshold in 48% of cases [14]. Another study found that the failure rate was 80% [15]. These studies concluded that pulse CO-Oximetry failed to warn clinicians of anemia. However, setting one fixed threshold value for all patients was inappropriate because SpHb overestimated the true Hb concentration with various errors in these studies. No study has set the decrease in SpHb as a threshold for the management of anemia.

Although hemodilution, active blood loss [10] and rapid transfusion [16] may influence the correlation between ΔSpHb and ΔCoOxHb, prior studies have found that the trend in SpHb is a good indicator of the change in Hb concentration when ΔCoOxHb is > 1 g/dl [10, 17, 18]. The consistency of the trending direction reached 86% ~ 95.4% [7, 18]. ΔSpHb was found to be positively related to ΔCoOxHb with *r* = 0.76, 95% CI = 0.57, 0.86 [7], which was consistent with our findings (*r* = 0.69). Decreases in SpHb probably warn of substantial changes in patients’ Hb in a timely manner in dynamic situations. However, only a small minority of SpHb studies have evaluated trend accuracy, and the triage role of the trend in SpHb has not been evaluated.

**Fig. 5:**
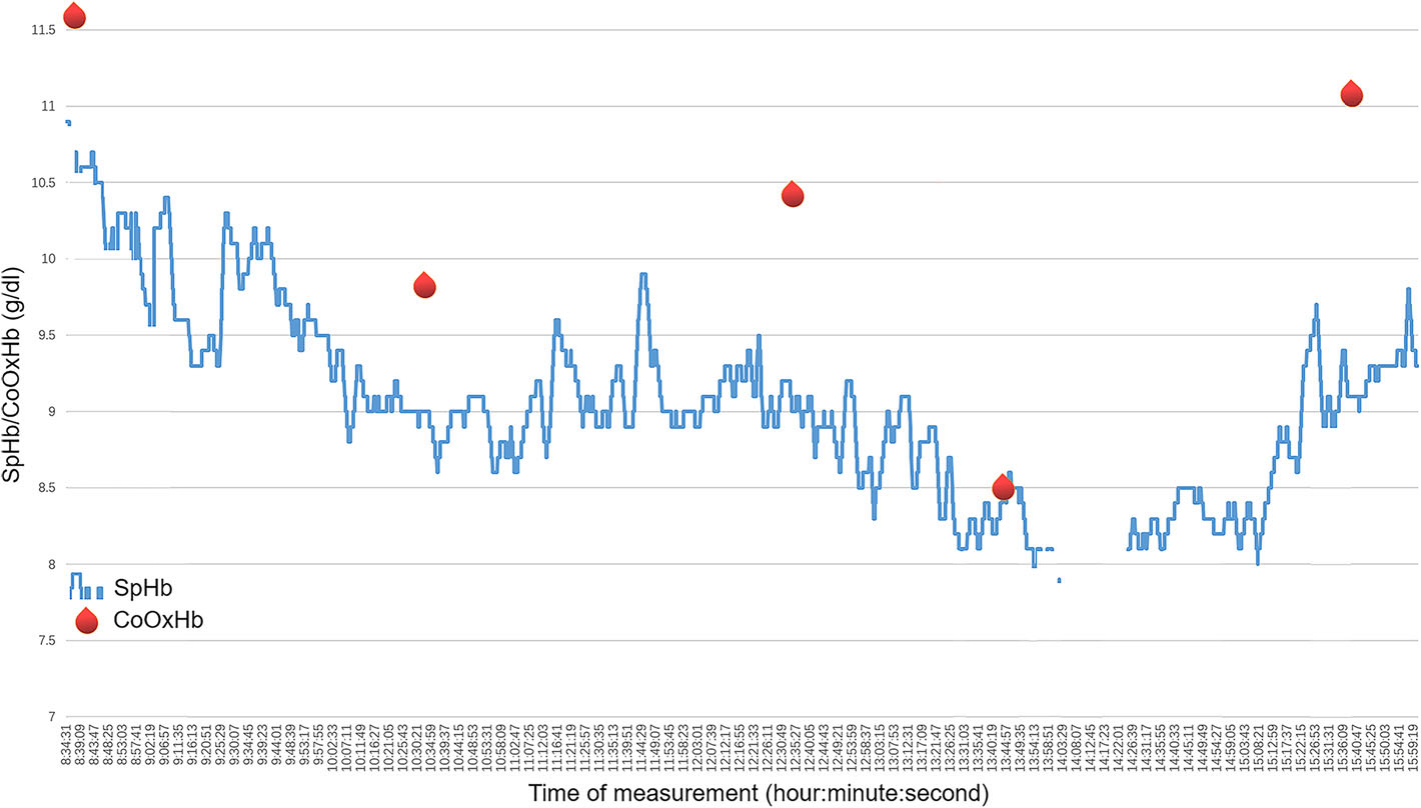
Application of SpHb monitoring in our study. A diagram showing the trends in SpHb (generated by the Pulse CO-Oximetry) and intermittent CoOxHb measurements taken in one patient in our study. Red blood drops represent invasive CoOxHb measurements. The blue wavy line represents the continuous monitoring of SpHb during surgery

We demonstrated the consistency between the trends in SpHb and CoOxHb in detail. The application of SpHb monitoring is shown in Fig. 5. Although there were various gaps existed between SpHb and the corresponding CoOxHb (indicating limited accuracy), the directions and amplitudes of the changes in SpHb were quite similar to those in CoOxHb. An interruption in the display due to poor peripheral perfusion also warned clinicians of serious anemia. The method used to evaluate the ability to track the trend in SpHb needs to be redefined. As a review suggested, an acceptable SpHb trend accuracy analysis does not require SpHb changes identical to invasive Hb changes but instead seeks to find directional agreement [3]. The stability of patients’ Hb levels can be determined by appropriate interpretation of the continuous trend in SpHb.

A novel method that takes full advantage of the trend in SpHb for the detection of anemia is described in this study. We found that timing the diagnostic blood draws according to changes in SpHb increased the PPV for anemia. Some studies that aimed to determine the PPV of SpHb changes compared to that of Hb changes reported PPVs only of 49–80% [10, 19]. However, in those studies, reference measurements were performed at the clinicians’ discretion, which might lead to a relatively low prevalence of a substantial decline in Hb and a decreased PPV for the trend in SpHb. The continuous trend in SpHb has not been fully exploited in clinical settings. In this study, a decrease in SpHb was set as the warning threshold and was able to more appropriately select the timing of invasive Hb measurements during surgery than clinicians. The trend in SpHb may play an active role in intraoperative transfusion decision-making in the future.

Although no patients received delayed treatment in the SpHb monitoring group, some arguments suggest that the change in SpHb may be delayed [14]. However, monitoring blood loss continuously and closely during long-term clinical work and estimating anemia accurately are difficult for doctors. Even a delayed SpHb may detect a change in anemia earlier than clinicians and enable doctors to focus on other important work. Therefore, setting the change in SpHb as an indication of intraoperative anemia should be encouraged.

In this study, no differences were found in intraoperative transfusion units or postoperative anemia between groups. Some studies found that intraoperative SpHb monitoring could save transfusion units [16], even reduce medical costs [20]. Although medical consumables such as sensors increase the expense, SpHb monitoring could save the cost of blood packaging and preservation by preventing unnecessary transfusions, leading to a reduced total cost. In fact, quantifying the improvement in patient outcomes due to use of a novel diagnostic method is extremely difficult, because other multiple therapeutic interventions may be performed at the same time. However, a timely Hb test result is an essential step in making a reasonable transfusion decision. Pulse CO-Oximetry may be a useful intraoperative monitor worth advocating based on clinical and economic aspects. More studies are needed to investigate the value-added benefits of SpHb monitoring in blood management.

## Limitations

One of the shortcomings of this study was the relatively high number of patients with a sufficiently large intraoperative blood loss volume to lead to anemia, which could have resulted in an overestimation of the SpHb monitoring PPV in the assessment of the decrease in CoOxHb. However, in this randomized trial, the PPV of the clinicians’ perceptions was also overestimated. Therefore, we could draw the conclusion that SpHb monitoring performed better in patients with a higher risk of blood loss than clinicians’ perceptions. Second, stratification analyses for various factors that affected accuracy were not performed in this study. More studies are needed to evaluate the triage role of SpHb monitoring in various clinical scenarios. Third, other investigators found that SpHb could reduce the number of transfusion units used, but this study did not influence the transfusion decision; therefore, that aspect requires further investigation.

## Conclusions

This study was the first diagnostic randomized controlled trial to explore the triage role of Pulse CO-Oximetry in the intraoperative detection of anemia. We found that the trend in SpHb could detect a decrease in Hb in dynamic situations and indicate the appropriate timing for further Hb measurements.

## Abbreviations

CoOxHb: CO-oximetry hemoglobin
POD: Postoperative day
PPV: Positive predictive value
SpHb: Continuous total hemoglobin
